# Ivermectin as an endectocide may boost control of malaria vectors in India and contribute to elimination

**DOI:** 10.1186/s13071-021-05124-3

**Published:** 2022-01-10

**Authors:** Sundus Shafat Ahmad, Manju Rahi, Poonam Saroha, Amit Sharma

**Affiliations:** 1 Indian Council of Medical Research (ICMR)–National Institute of Malaria Research, New Delhi, India; 2 Indian Council of Medical Research, New Delhi, India; 3 International Centre for Genetic Engineering and Biotechnology, New Delhi, India; 4 Academy of Scientific and Innovative Research (AcSir), Ghaziabad, India

**Keywords:** Endectocide, Ivermectin, Malaria elimination, Vector control

## Abstract

Malaria constitutes one of the largest public health burdens faced by humanity. Malaria control has to be an efficient balance between diagnosis, treatment and vector control strategies. The World Health Organization currently recommends indoor residual spraying and impregnated bed nets as two malaria vector control methods that have shown robust and persistent results against endophilic and anthropophilic mosquito species. The Indian government launched the National Framework for Malaria Elimination in 2016 with the aim to achieve the elimination of malaria in a phased and strategic manner and to sustain a nation-wide malaria-free status by 2030. India is currently in a crucial phase of malaria elimination and novel vector control strategies maybe helpful in dealing with various challenges, such as vector behavioural adaptations and increasing insecticide resistance among the *Anopheles* populations of India. Ivermectin can be one such new tool as it is the first endectocide to be approved in both animals and humans. Trials of ivermectin have been conducted in endemic areas of Africa with promising results. In this review, we assess available data on ivermectin as an endectocide and propose that this endectocide should be explored as a vector control tool for malaria in India.

**Figure:**
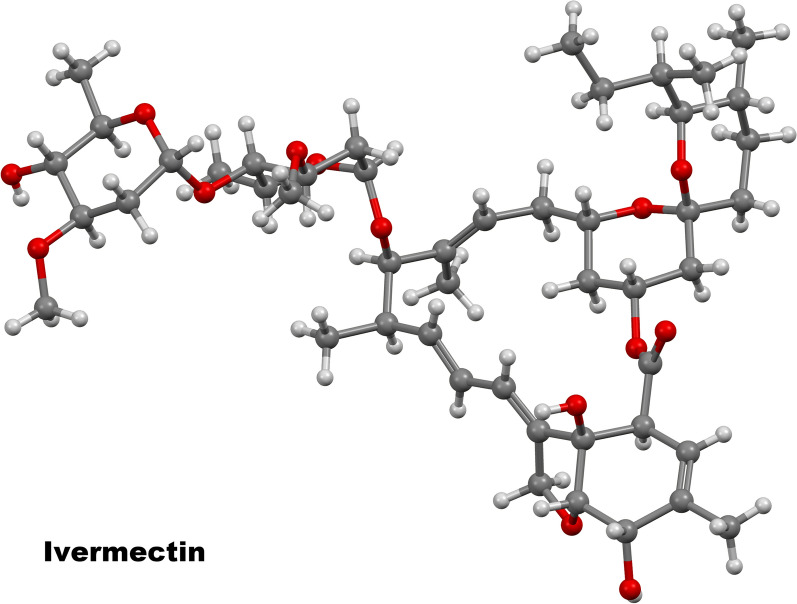

## Background

Malaria is a significant public health issue in India, with a complex heterogeneity due to the presence of six major anopheline vector species across different eco-geographical regions of the country [1]. In recent years, these vectors have shown a change in distribution and behaviour. The estimated number of malaria cases worldwide in 2019, as reported by the World Health Organization (WHO) in the World Malaria Report 2020, was 229 million, with the South-East Asia region accounting for 3% of all malaria cases worldwide [2]. Although the WHO reported that India had achieved the highest reduction in malaria cases of all countries tabulated (i.e. from 20 million cases in 2000 to 5.6 million in 2019), it still accounted for  ~ 86% of all malaria associated deaths in South Asia [2]. These losses can be avoided as malaria is considered to be a preventable and curable disease. Vector control has always been a vital component of malaria control strategies. Long-lasting impregnated insecticide bed nets (LLINs) and indoor residual insecticide sprays (IRS) are the backbone of vector control strategies and have contributed to reducing the burden of malaria [3]. Between 2000 and 2015, IRS and LLINs have collectively accounted for a decline of  ~ 78% in malaria cases in endemic regions of Africa [4].

The increasing resistance of *Anopheles* spp. to currently used insecticides is an impending threat to malaria management programmes [5, 6]. In addition, changes in vector behaviour to earlier biting and outdoor biting patterns have been documented in India and elsewhere [5, 6]. Such changes in vector behaviour impact both LLINs and IRS [6]. Many vector control tools have been tested in research settings such as insecticide-impregnated hammocks, insecticide-treated clothing, footwear, strips, wall linings, wall paints, spatial/airborne repellents, topical repellents, mosquito traps, attractive toxic sugar baits and endectocides [7, 8]. Endectocides are drugs with both endoparasitocidal and ectoparasitocidal activity, and they are widely used in veterinary medicine. One such anti-parasitic agent is ivermectin which is also approved for human use [3].

## Retrieval of information

Available data on ivermectin as an endectocide was retrieved from published medical and veterinary entomology documents. We performed an online search of the PubMed, Cochrane Library, Google and Google Scholar bibliographic databases for scientific papers published from 2010 until the present, using a combination of the following search terms: “vector control”, “vector control methods”, “malaria”, “endectocide”, “ivermectin” and “*Anopheles*”. The search identified 15 completed trials that satisfied the search criteria for this review: seven human trials and eight cattle trials (see Tables 1, 2). Data from ongoing trials were retrieved from the Malaria Eradication Scientific Alliance. Treatment guidelines and protocols were retrieved from official websites of the National Vector Borne Diseases Control Programme and WHO.

**Table 1:** Major ivermectin studies in humans

Trial name/first author of study(year) [reference]	Dosage of ivermectin	Results: mortality of mosquito
ACTIVE [24] 2013 Burkina Faso	200 µg/kg as a single dose or 200 µg/kg for 2 days as two doses or placebo; all administered with artemether-lumefantrine	*An. gambiae* Mortality of mosquito in 3 days post blood meal (one dose of Ivermectin): 33% Mortality of mosquito in 3 days post blood meal (two doses of Ivermectin): 31% Controls: 6% mortality in 3 days (difference was significant) Mortality of mosquito in 10 days post blood meal (one dose of Ivermectin): 59% Mortality of mosquito in 10 days post blood meal (2 doses of Ivermectin): 66% Controls: 21% mortality in 10 days (difference was significant) *An. funestus* Mortality of mosquito in 3 days post blood meal (one dose of Ivermectin): 33% Mortality of mosquito in 3-days post blood meal (two doses of Ivermectin): 22% Controls: 3% mortality in 3 days (difference was significant) Mortality of mosquito in 10 days post blood meal (one dose of Ivermectin): 40% Mortality of mosquito in 10 days post blood meal (two doses of Ivermectin): 51% Controls: 5% mortality in 10 days (difference was significant)
RIMDAMAL [15] 2015 Burkina Faso	First dose of 150–200 µg/kg single dose with 400 mg albendazole plus five further similar ivermectin doses at 3-week intervals	Frequently repeated mass administration of ivermectin during the malaria transmission season led to significant reduction in malarial episodes in children
IVERMAL [25] 2015 Kenya	300 µg/kg for 3 days or 600 µg/kg for 3 days or placebo; all administered with dihydroartemisinin-piperaquine	3-Day ivermectin treatment at either of the doses reduced mosquito survival for at least 28-days-post feeding
Derua [26] 2015 Tanzania	150–200 μg/kg	Mortality of mosquito in 3 days post blood meal: 66.2% Mortality of mosquito in 9 days post blood meal: 95% Significance not reported
Sampaio [27] 2016 Brazil	200 µg/kg	Ivermectin treatment reduces mosquito survivorship by 4 h to 14 days
Kobylinski [28] 2017 Thailand	200 µg/kg	Ivermectin was lethal to dominant GMS Anopheles malaria vectors and inhibited sporogony of *P. vivax* at safe human relevant concentrations
Mekuriaw [14] 2019 Ethiopia	Single oral dose of 12 mg	Significant higher mortality of mosquitos on days 1 and day 4 reported

**Table 2:** Major ivermectin studies carried out in cattle

Study/first author of study(year) [reference]	Dosage of ivermectin	Mortality of mosquitos
Fritz [29] 2009 Kenya	600 µg/kg once subcutaneously	Mortality of mosquitos in 3 days post blood meal, (feeding done one day after ivermectin treatment): 100% Controls: 10% (significance not reported) Mortality of mosquitos in 3 days post blood meal (feeding done 13 days after ivermectin treatment): 62% Mortality of mosquitos in 9 days post blood meal (feeding done 13 days after ivermectin treatment): 88%, Controls: 10–38% (significance not reported)
Naz [17] 2013 Pakistan	200 µg/kg once subcutaneously	*An. culicifacies* Mortality of mosquito in 3 days post blood meal: 65% Controls: 9% Mortality of mosquito in 9 days post blood meal: 80% Controls: 17% *An. stephensi* Mortality of mosquito in 3 days post blood meal: 80% Controls: 10% Mortality of mosquito in 9 days post blood meal: 80% Controls: 25%
Pooda [30] 2015 Burkina Faso	200 µg/kg injected	Reduction in mortality of mosquitos by 75% in the third week and by 45% in the fourth week post ivermectin treatment
Poche [18] 2015 Kenya	100–200 µg/kg orally	Mortality of mosquito in 3 days post blood meal: 45–63% Mortality of mosquito in 9 days post blood meal: 65–94%
Lyimo [31] 2017 Tanzania	200 µg/kg once subcutaneously	Survival and fecundity of *An. arabiensis* were reduced by 52.5% and 64.6%, respectively
Chaccour [19] 2018 Tanzania	5 subcutaneous implants of 23 mg each, tested over 40 weeks	Significant increased mortality of mosquitos in 3 days and 10 days after blood meal (significant difference)
Cramer [16] 2021 Vietnam	200 µg /kg once subcutaneously	Ivermectin treatment significantly reduced survivorship of *An. dirus* up to 20 days and *An. epiroticus* up to 8 days
Makhanthisa [32] 2021 South Africa	200 µg/kg injected	Significant increased mortality of mosquitos on day 7, 13 and 21 post ivermectin treatment and also lead to reduced egg production

## Ivermectin use in malaria

Ivermectin is a semi-synthetic avermectin derivative that was first licensed in 1981 as a veterinary drug and then approved in 1987 for use in humans due to its activity against the parasites of* Onchocerca* spp. [9]. It is currently authorized for the treatment of headlice, lymphatic filariasis, onchocerciasis, strongyloidiasis and scabies [10]. Over the past 30 years it has been found to be a remarkably potent insecticide and anthelmintic, especially against filarial worms [3]. The use of ivermectin for malaria vector control was first suggested in 1985, following publication of a study showing that this drug killed *Anopheles stephensi* in in vitro tests [9]. The basis of ivermectin-based malaria control is that it reduces the survival of mosquitoes that feed on human or cattle populations previously administered with ivermectin. Ivermectin has mosquitocidal activity, and its administration to humans and/or livestock reduces the lifespan of mosquitoes irrespective of biting patterns or host preference. Consequently, this drug has the potential to complement the existing toolbox of malaria vector control measures [11].

Recognizing the potential of ivermectin, the WHO Malaria Policy Advisory Committee (MPAC) in its technical consultation reviewed the available data on ivermectin in 2016. The MPAC put forward a policy recommendation that for ivermectin to be considered of public health relevance, at least 20% reduction in clinical malaria incidence has to be demonstrated at least 1 month post treatment with one round of mass administration of ivermectin [12]. However, the concept of using ivermectin as an endectocide against malaria vectors does pose an ethical conundrum. This drug is not given as a prophylaxis or as a malaria treatment, but as a vector control method targeting mosquitoes for the larger public good.

There have been several studies in Africa and Asia that have deployed ivermectin in humans as a vector control tool (Table 1). The most common dosage used in these trials was 150–200 µg/kg as single oral dose or up to 400 µg/kg in multiple doses [13]. Mass ivermectin treatment in human populations targets anthropophilic *Anopheles* vectors and endophagic *Anopheles*. Mekuriaw et al. [14] reported in 2019 that the mortality of mosquitoes fed on ivermectin-treated blood was significantly higher than that of the controls (13.8 vs 3.7%). These authors also reported that the fecundity of the ivermectin-treated mosquito populations was lower than that of the controls [14]. Most of the studies on the mosquitocidal activity of ivermectin have been conducted under controlled laboratory conditions. In contrast, a randomized controlled trial (RIMDAMAL) was conducted by Foy et al. [15] in 2015 in the field, and it demonstrated that children who had received ivermectin had reduced number of malaria episodes as compared to the control group and there was an overall reduction in malaria transmission. There was a reduced incidence of malaria episodes in the intervention arm (648 episodes in 327 children; average of 2 episodes per child) in comparison to the control arm (647 episodes in 263 children; average 2.5 episodes per child) [15].

There are a number of ongoing large-scale trials of ivermectin as an endectocide for malaria control, including RIMDAMAL II (Burkina Faso), MATAMAL(Guinea-Bissau), BOHEMIA (Tanzania- Mozambique), REACT (Burkina Faso and Côte d'Ivoire) and MASSIV (The Gambia) [13]. The effect of ivermectin when administered at a dose of 200 µg/kg to cattle targeting zoophagic mosquitoes has also been studied (Table 2). For cattle, there are a variety of administration modes/formulations, including subcutaneous, injectable and implantable (long-release solutions) [13]. A major trial by Cramer et al. [16], carried out by the University of Vietnam and University of Massachusetts, was based on zooprophylaxis-aided ivermectin-based vector elimination (ZAIVE) in 2021. The trial was carried out in Vietnam which has a significant problem with mosquitoes in forested areas. In this study, the mosquitoes were fed on cattle that had been injected subcutaneously with 0.2 mg/kg ivermectin (intervention arm) or not (control arm) and then these mosquitos were collected from both the intervention and control sites to analyse anopheline populations prior to and post dosing of cattle with ivermectin [16]. The mortality of the mosquitoes was checked for up to 30 days post feeding [16]. The results showed, for the first time in South-East Asia, that cattle treated with ivermectin at standard veterinary dosages led to reduced survival of two important malaria vectors, *Anopheles epiroticus* and *Anopheles dirus* [16]. It also determined that an adequate population of livestock dosed with ivermectin in peri-domestic situations would have a significant impact on anopheline numbers [16]. Reports by Naz et al. [17] in 2013 and Poche et al. [18] in 2015 also showed significant mortality among mosquitoes (80–95%) fed on ivermectin-treated cattle.

There are advantages to administering ivermectin in cattle over human administration: (i) ivermectin administration in cattle targets a wide array of zoophagic *Anopheles*, thus reducing malaria transmission; (ii) it is easier to obtain institutional/regulatory agency approval for trials in animals compared to humans; (iii) long-lasting formulations and a wider diversity of formulations (injectable and implants) with longer drug half-life can be used in cattle. For example, Chaccour et al. [19] showed that an ivermectin formulation when implanted in cattle was successful in delivering medication for a duration of 6 months. This reduced malaria transmission and also showed a (iv) collateral benefit of increasing livestock weight gain and milk yield, which in turn helps the community [20].

India is predominately an agricultural society, with 70% of its population living in rural settings. In this setting, humans commonly cohabit with cattle and farm animals. Also, 70% of malaria in India is attributed to *Anopheles culicifacies* [1], a zoophilic species. Therefore, there is an increased probability of close contact between the general human population and animals and under such conditions, ivermectin administration to cattle populations will not only have a beneficial effect on suspectible human populations, but anthropophilic mosquitoes will also be targeted. In addition, an increased overall impact can be achieved as the resulting reduction in *Anopheles* density can enhance the action of commonly used vector control tools like LLINs and IRS, thereby augmenting their impact [11].

**Fig. 1:**
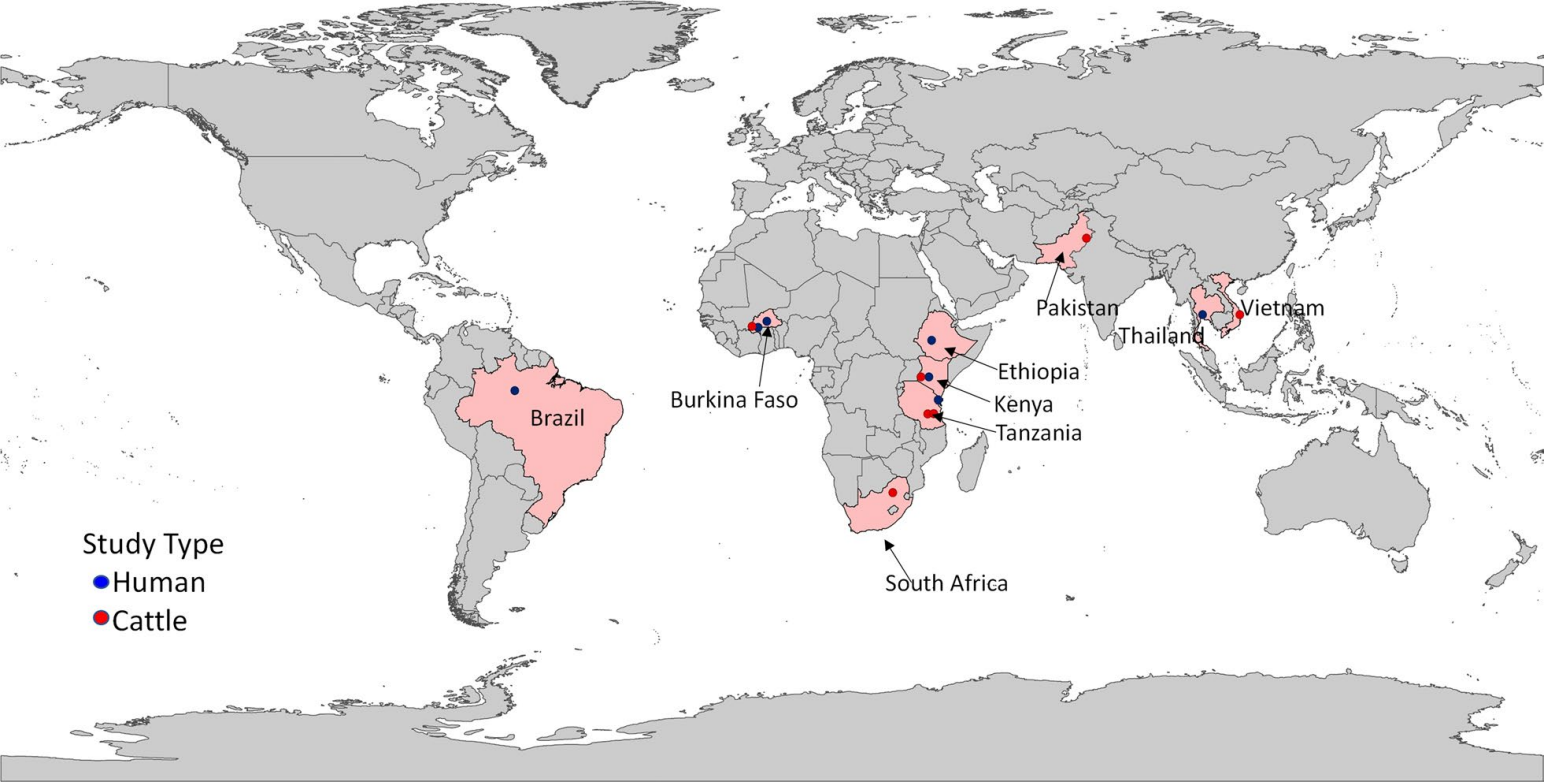
Studies carried out worldwide using ivermectin for malaria vector control (2013–2021)

**Fig. 2:**
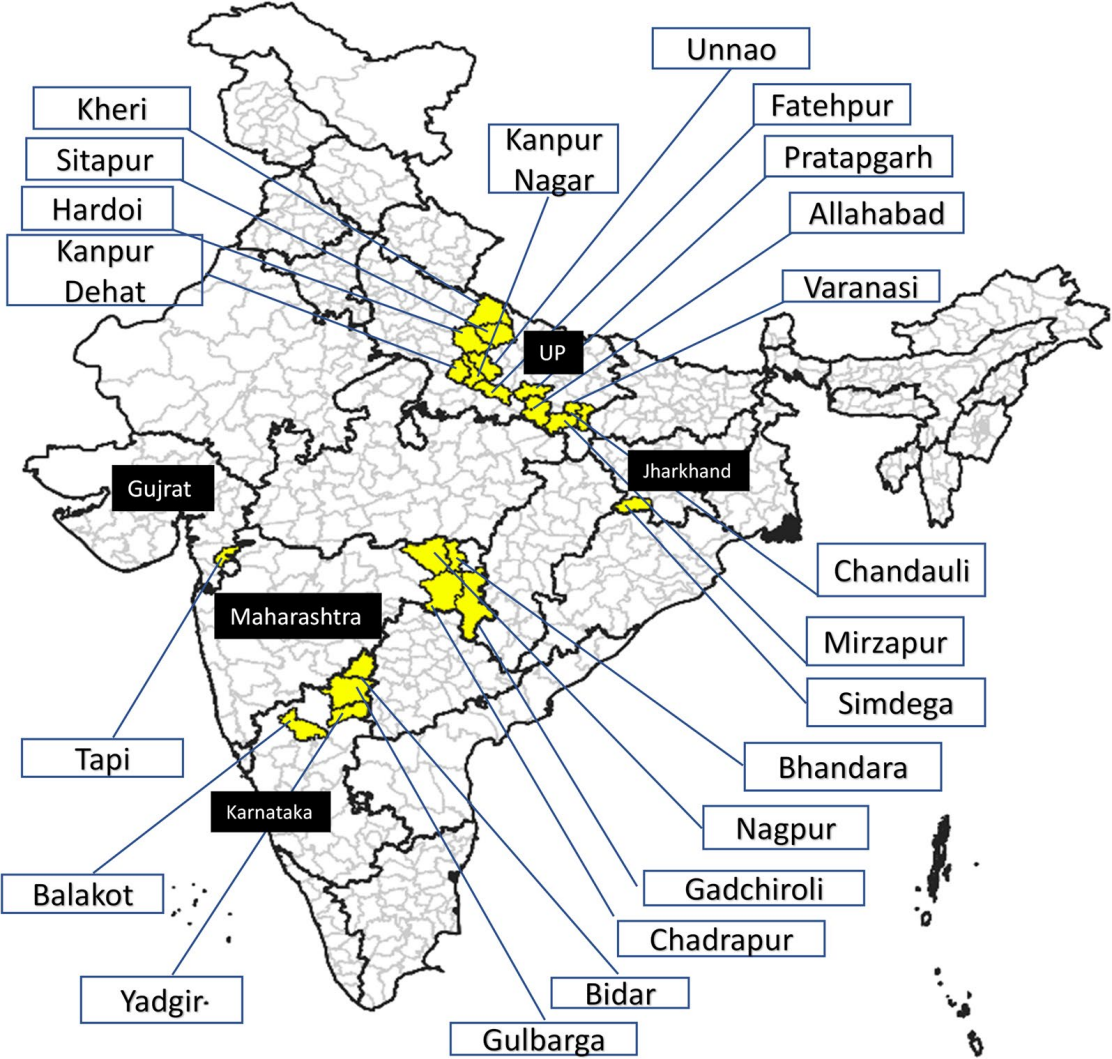
Districts in India where which ivermectin is distributed in the National Programme to Eliminate Lymphatic Filariasis

 Figure 1 shows a global map of studies on the use of ivermectin as endectocide in humans and cattle. It should be noted that ivermectin is not a new drug to the Indian public health setup. Since 2018, the Indian National Programme to Eliminate Lymphatic Filariasis has incorporated ivermectin as the third drug along with albendazole and diethylcarbamazine as a preventive mass chemotherapy. The triple therapy is successfully deployed in 21 districts across the country (depicted in Fig. 2) [21].

Despite achieving a reduction in malaria burden in recent years, India reported 181,831 malaria cases in 2020 [21]. As noted above, India is primarily a rural and agricultural state and houses 37% of the world’s livestock [22]. In 2020, the state of Odisha accounted for 23% of all malaria cases in India, followed by the state of Chhattisgarh which shouldered 20% [5]. Despite effective intervention tools, malaria burden continues to be high in certain districts of India, suggestive of residual malaria transmission wherein despite adequate coverage with effective vector control tools like ITNs and/or IRS malaria cases remain high [21].

## Steps to testing and evaluating ivermectin as endectocide in India

Although ivermectin is an established drug in India, its usage targeting Indian malaria vectors needs to be researched. First, the susceptibility of Indian anopheline species to ivermectin is not known and needs to be established—especially for *Anopheles culicifacies* which is responsible for > 70% malaria transmission in India [1]. Second, the 50% lethal dose (LD50) and 90% lethal dose (LD90) for Indian malaria vectors needs to be determined followed by preclinical studies [23]. As a subsequent step, ivermectin could then be tested in cattle and human populations in malaria endemic areas in India for its impact on malaria vectors [12]. An existing mass drug administration (MDA) programme using ivermectin for lymphatic filariasis can be leveraged while planning ivermectin trials for malaria. The mosquitocidal effect of ivermectin on insecticide-resistant *Anopheles* spp. can also be evaluated as a potential advantage [12]. In accordance with WHO suggestions, study designs could include: (i) observational studies in locations where ivermectin MDA is already underway against lymphatic filariasis; (ii) cluster randomized controlled trials that estimate the benefits of ivermectin in addition to core vector control strategies and management of cases; and (iii) before and after studies of ivermectin MDA in control and intervention sites [12].

For human trials, it must be noted that subjects will be consuming the drug for the benefit of others, and participating communities will need education and explanations to understand this concept. Also, the communities need to be made aware that it would not be a prophylactic and therapeutic option for malaria. Ivermectin will be a supplementary measure and not a replacement of existing vector control methods. If proven successful via testing on Indian vectors, the deployment of ivermectin can be initially limited to certain hotspots that are experiencing persistent malaria outbreaks. India seems closer to malaria elimination than ever before, and yet we need newer vector management tools to cover regions with persistent malaria where conventional tools fall short in effective malaria control.

## Conclusion

Insecticide resistance and changing behaviour of the malaria vectors are crucial challenges to vector control strategies which can potentially weaken the drive towards malaria elimination in India. Among other novel methods/tools, the use of ivermectin as an endectocide holds promise, as shown in international animal and human trials. India is yet to explore the use of ivermectin as a mosquitocidal agent. This is an opportune time to assess ivermectin in Indian malaria vectors in a graded manner, beginning with testing the susceptibility of Indian vectors to ivermectin, followed by preclinical studies and then clinical studies in cattle and humans. The case scenario for the endectocide could be prioritized in consultation with the national programme and with sufficient sensitization and education of the communities. After these steps have been carried out, the possibility of deploying ivermectin as a vector control tool can be envisaged.

## Data Availability

This paper does not contain original data.
